# Camrelizumab plus rivoceranib versus sorafenib as first-line therapy for patients with unresectable hepatocellular carcinoma: a cost–utility analysis in China and the United States

**DOI:** 10.3389/fphar.2025.1404389

**Published:** 2025-05-01

**Authors:** Qiuling Zhao, Yimin He, Zilin Nian, Yongjian Huang, Ruyi Huang, Lijun Lai, Lin Yang

**Affiliations:** ^1^ Clinical Oncology School of Fujian Medical University, Fujian Cancer Hospital, Fuzhou, Fujian, China; ^2^ School of Pharmacy, Fujian Medical University, Fuzhou, Fujian, China

**Keywords:** hepatocellular carcinoma, camrelizumab, rivoceranib, sorafenib, cost–utility analysis

## Abstract

**Objective:**

Camrelizumab plus rivoceranib (camr-rivo) has been shown to significantly improve overall survival (OS) in patients with unresectable or advanced hepatocellular carcinoma (HCC) in the CARES-310 trial. However, the cost-utility of this treatment remains unclear. Therefore, this study evaluated the cost–utility of camr-rivo versus sorafenib as a first-line systemic therapy for patients with unresectable or advanced HCC from the perspectives of the Chinese healthcare system and the United States (US) payers.

**Methods:**

Based on the CARES-310 trial, a partitioned survival model was constructed to estimate economic costs and health outcomes over a 10-year lifetime horizon. Drug costs were obtained from the public database, Red Book, and relevant literature. Health utility values were derived from the literature. One-way and probabilistic sensitivity analyses were performed. The willingness-to-pay (WTP) threshold was $36,627.25/QALY in China and $150,000.00/QALY in the United States.

**Results:**

Camr-rivo yielded an additional 0.34 quality-adjusted life years (QALY) compared to sorafenib for patients with unresectable or advanced HCC. The incremental costs in China and the United States were $4,762.10 and $92,700.49, respectively, and the incremental cost–utility ratios (ICURs) were $14,174.40/QALY and $272,852.59/QALY, respectively. Sensitivity analyses indicated that the cost of rivoceranib and camrelizumab had the greatest impact on the ICUR in China and the United States. Scenario analyses showed that a price reduction of approximately 30% for camrelizumab and rivoceranib could make camr-rivo a cost-utility option in the United States.

**Conclusion:**

At the set WTP threshold, camr-rivo is a cost–utility treatment strategy compared to sorafenib as a first-line therapy for patients with unresectable or advanced HCC in China but not in the United States.

## 1 Introduction

Hepatocellular carcinoma (HCC) is a major type of liver cancer, ranking sixth in incidence and third in mortality worldwide (Bray et al., 2018). In 2020, 410,038 new cases and 391,152 deaths were reported in China, accounting for nearly half of the global incidence and mortality rates of HCC (The Global Cancer Observatory, 2021). In the United States (US), the 5-year survival rate for HCC is extremely low (only 21%), ranking second only to pancreatic cancer (Siegel et al., 2023). HCC imposes a considerable disease and economic burden on the global healthcare system. Therefore, providing a novel and effective therapy for patients with HCC is an imperative clinical requirement.

Most patients with HCC are diagnosed with unresectable or advanced HCC (Liu et al., 2021a). Systemic therapy has become the standard treatment for such patients. As the first approved small-molecule targeted tyrosine kinase inhibitor (TKI), sorafenib has been the only first-line systemic treatment for advanced HCC for a decade prior to 2018 (National Comprehensive Cancer Network, 2023). Since then, several new TKIs, including donafenib and lenvatinib, and vascular endothelial growth factor (VEGF) inhibitors, such as bevacizumab, have been approved as first-line treatments (2022, Qin et al., 2023), but their clinical efficacy has not been satisfactory. With the rapid development of immunotherapy, the systemic treatment of HCC has taken a crucial step forward. Programmed death receptor 1 (PD-1) or programmed death ligand 1 (PD-L1) antibodies, including pembrolizumab, sintilimab, tislelizumab, and atezolizumab, played essential roles increasingly in the HCC clinical practice.

PD-1/PD-L1 inhibitor-based combination therapy has shown promising efficacy and has been recommended as the first-line treatment for advanced HCC, according to several guidelines (Heimbach et al., 2018). Inspired by atezolizumab plus bevacizumab, sintilizumab plus bevacizumab analogue was the preferred recommendation as the first-line treatment regimen (Chinese Society of Clinical Oncology, 2021). Recently, camrelizumab plus rivoceranib (camr-rivo) was approved by the Chinese National Medical Products Administration (NMPA) on January 31, 2023, and was the first globally approved combination of a PD-1 inhibitor with a small-molecule antiangiogenic drug for treating unresectable or advanced HCC.

A randomized, open-label, international phase III trial (CARES-310) demonstrated the efficacy and safety of camr-rivo versus sorafenib in advanced metastatic or unresectable HCC (Qin et al., 2023). The median overall survival (mOS) was significantly prolonged in patients receiving combination therapy compared to sorafenib, reaching 22.1 months, which was the longest duration observed in patients with unresectable or advanced HCC among all systemic combination therapies in phase III trials. The results also showed that camr-rivo significantly prolonged progression-free survival (PFS) compared to sorafenib (median PFS 5.6 months vs 3.7 months, hazard ratio [HR] 0.52). Thus, based on its effectiveness, camr-rivo offers a new, more effective choice for patients with HCC. However, considering cost utility in medical decision-making is crucial for optimizing the allocation of limited healthcare resources. Despite the significant improvement in median OS and PFS observed with camr-rivo in patients with HCC, there is a lack of economic evidence to assess their affordability, and the lack of information hampers decision-makers’ ability to make well-informed choices. Therefore, the objective of this study was to conduct a comparative analysis of the cost–utility of camr-rivo and sorafenib as first-line treatments for patients with unresectable HCC in China and the US and establish a foundation for the development of rational and effective treatment strategies.

## 2 Patients and treatments

Patients were randomly assigned in a 1:1 ratio to receive either camr-rivo or sorafenib monotherapy. According to the CARES-310 trial, the eligible patients were aged ≥18 years, with histopathologically or cytologically confirmed HCC, Barcelona Clinic Liver Cancer stage B or C disease, and were either unresectable or had progressed after surgical or locoregional therapy; they had not previously received treatments, had Child-Pugh class A liver function, an Eastern Cooperative Oncology Group (ECOG) performance status of 0 or 1, and one or more measurable lesions according to the Response Evaluation Criteria in Solid Tumors version 1.1 (RECIST 1.1).

Patients received 200 mg of camrelizumab intravenously every 2 weeks plus 250 mg of rivoceranib orally once daily (camr-rivo) or sorafenib 400 mg orally twice daily, until unacceptable toxicity or disease progression occurred. A total of 90 (33.1%) patients in the camr-rivo group and 130 (48.3%) patients in the sorafenib group were treated with second-line therapy after disease progression. In order to better simulate the actual second-line treatment that better fits the real world, we chose second-line regimens for which the largest proportion of patients was treated, including lenvatinib, camrelizumab, sorafenib, regorafenib, sintilizumab, rivoceranib, and capecitabine. The proportion of patients receiving each second-line regimen and the cost of second-line therapies in China and the US are detailed in Supplementary Table S1. The specific course of the second-line treatment is provided in Supplementary Table S2. We assumed that the patients received second-line therapy until disease progression or the occurrence of unacceptable toxicity.

## 3 Methods

### 3.1 Model structure

**FIGURE 1:**
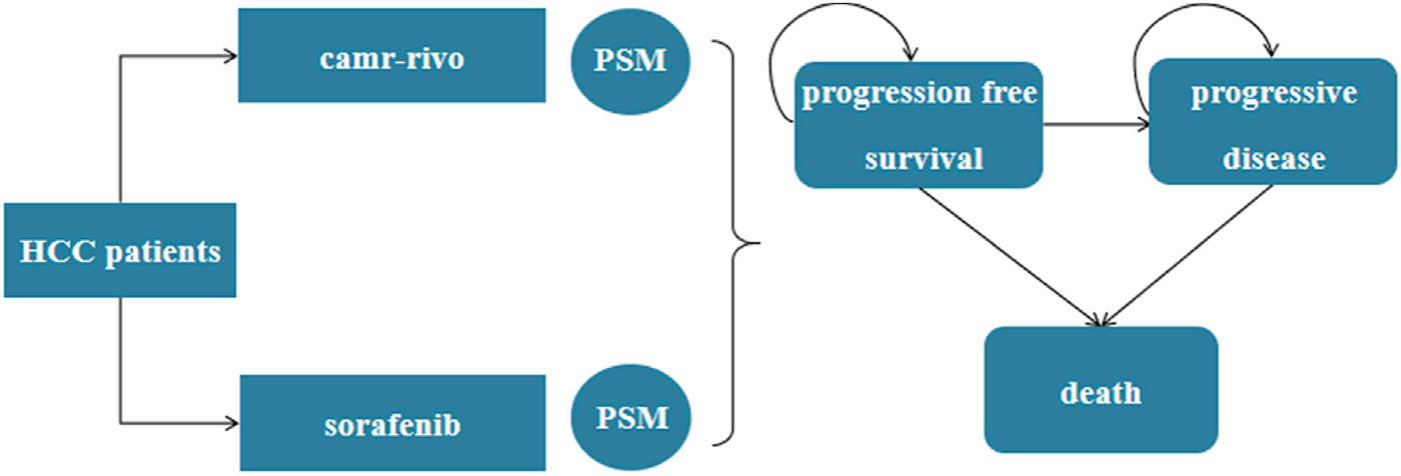
The partitioned-survival model for unresectable or advanced HCC. HCC, hepatocellular carcinoma; PSM, partitioned-survival model; camr-rivo, camrelizumab-rivoceranib.

A three-mutually exclusive status partitioned-survival model (PSM) was constructed using TreeAge Pro 2022 software (TreeAge, Williamstown, MA), including PFS, progressive disease (PD), and death (Figure 1). The proportion of patients in each health status at time *t* was estimated based on PFS and OS curves. At a given time point *t*, the PFS curve depicts the proportion of patients who remain free from disease progression, whereas the OS curve indicates the proportion of patients who are still alive. Therefore, the proportion of patients in the PD state at time *t* should be the difference between the two curves (TREEAGE SOFTWARE, INC, 2023). Patients can transition from PFS to PD status, but they cannot return to PFS status (Williams et al., 2016).

The model used 28-day cycles to match treatment patterns. China’s Pharmacoeconomic Guidelines (2020) recommend modeling until the cohort survival probability falls below 1% (Chinese Pharmaceutical Association, 2020). Our extrapolated survival curves showed <1% survival at 10 years for both arms; thus, the time horizon was 10 years, about a lifelong time. The main model outputs were quality-adjusted life years (QALYs) and incremental cost-utility ratio (ICUR). The willingness-to-pay (WTP) threshold was set as $36,627.25/QALY (three times the gross domestic product *per capita* by 2022) in China and $150,000.00/QALY in the US (Neumann et al., 2014; Chinese Pharmaceutical Association, 2020). The model parameters adopted a discount rate of 5% and 3% per year in China and the US, respectively.

This study followed the Consolidated Health Economic Evaluation Reporting Standards (CHEERS) reporting guidelines (Husereau et al., 2022) (Supplementary Table S6). Our study was exempted from institutional review board review and from obtaining informed consent because it was based on publicly available data and modeling techniques.

### 3.2 Clinical inputs

In PSM, the status of the simulated patient cohort was estimated by extrapolating the study data on cumulative probabilities of PFS and OS. First, WebPlotDigitizer software was used to digitize the Kaplan–Meier OS and PFS curves, and pseudo-individual patient data (IPD) were reconstructed. Then, in R (version 4.0.2) software, the Kaplan–Meier curves for each group were reconstructed (Patricia et al., 2012). Second, Weibull, gamma, Gompertz, and exponential distributions were fitted to the reconstructed individual data (Guyot et al., 2012). Finally, based on rigorous statistical criteria, such as the Akaike information criterion (AIC) and Bayesian information criterion (BIC), the Weibull distribution was selected as the optimal option for the OS curve of the camr-rivo and gamma distribution for other curves (Supplementary Table S3).

### 3.3 Costs and utility values

Costs were calculated from the perspectives of the Chinese healthcare system and the US payers; therefore, direct medical costs were calculated, such as drug costs, follow-up costs, including laboratory test cost, computed tomography (CT)/magnetic resonance imaging (MRI) examination cost, administration cost, end-of-life care cost, and treatment cost of grade ≥ 3 adverse events (AEs) (Wen et al., 2021; Kim et al., 2020; Parikh et al., 2017; Meng et al., 2022; Dong et al., 2022; Shu et al., 2023; Zhang et al., 2021; Zhou et al., 2022; Gu et al., 2019; Lin et al., 2021) (Supplementary Table S4). Due to the large proportion of patients with concurrent hepatitis B, the cost of anti-HBV drug therapy in patients with HCC, mainly the cost of entecavir, was also taken into account in the Chinese model. In addition, because the prices of camrelizumab and rivoceranib were not available in the US, the cost of atezolizumab in China replaced the cost of camrelizumab per cycle, and the price of lenvatinib replaced the price of rivoceranib in the US model. Drug prices were acquired from the Chinese Health Industry Data Center (yaozh.com), Red Book, and relevant literature. Health utility values were derived from previously published studies (Wen et al., 2021). In addition, disutility associated with AEs was also included (Liu et al., 2021b; Telford et al., 2019; Hoerger et al., 2010; Shao et al., 2022; Kim et al., 2020) (Supplementary Table S4). The probabilities of AEs in each group are listed in Supplementary Table S5. Although transient AEs typically recur during treatment cycles, our model simplified AE-related costs and disutilities to a one-time calculation in the first treatment cycle based on methodologies from cited pharmacoeconomic studies on HCC (Wu et al., 2019; Luo et al., 2022; Li et al., 2021). This approach aligns with previous studies, demonstrating that most grade ≥3 AEs in HCC systemic therapy occur early (within 1–2 cycles) (Wu et al., 2019). Single-cycle aggregation maintains model validity while reducing computational complexity (Li et al., 2021). All costs were converted into US$ 2023, with an exchange rate of $1 = 7.02. Details of the model parameters are listed in Table 1.

**TABLE 1:** Model parameters.

Parameters	Base-case value (Range^a^) in China	References	Base-case value (Range^a^) in US	References
Costs per cycle ($)				
Camrelizumab	734.17 (587.34–881.00)	yaozh, 2023	6151.31 (4921.05–7381.57)	yaozh, 2023
Rivoceranib	417.58 (334.06–501.08)	yaozh, 2023	8874.70 (7099.76–10649.64)	Kim et al. (2020)
Sorafenib	247.32 (197.86–296.78)	yaozh, 2023	18,649.12 (14,919.30–22378.94)	Micromedex Solutions, 2023
Second-line therapy costs in each group				
Camr-rivo	191.72 (153.38–230.06)	Qin et al., 2023; Micromedex Solutions, 2023; Kim et al., 2020; Parikh et al., 2017	4149.34 (3319.47–4979.21)	Qin et al., 2023, Micromedex Solutions, 2023; Kim et al., 2020, Parikh et al., 2017
Sorafenib	343.27 (274.62–411.92)	Qin et al., 2023; Micromedex Solutions, 2023; Kim et al., 2020; Parikh et al., 2017	4536.01 (3628.81–5443.21)	Qin et al., 2023; yaozh, 2023; Kim et al., 2020; Parikh et al., 2017
Anti-HBV therapy costs in each group				
Camr-rivo	16.40 (13.12–19.68)	yaozh, 2023	—	—
Sorafenib	15.75 (12.6–18.90)	yaozh, 2023	—	—
Follow-up costs ($/per time)				
Laboratory tests	168.76 (135.01–202.51)	Wen et al. (2021)	752.70 (601.60–903.24)	Wen et al. (2021)
CT/MRI examination	21.37 (17.10–25.64)	Meng et al. (2022)	155.94 (124.75–187.13)	Dong et al. (2022)
Administration	119.00 (95.20–142.80)	Shu et al. (2023)	135.52 (108.41–162.61)	Zhang et al. (2021)
End-of-life care	2047.45 (1637.96–2456.94)	Zhou et al. (2022)	31,343.71 (25,074.97–37612.45)	Zhang et al. (2021)
Treatment cost of AEs (grade ≥3) in each group				
Camr-rivo	206.24 (164.99–247.49)	Wen et al., 2021; Meng et al., 2022; Gu et al., 2019	3726.68 (2981.34–4472.02)	Wen et al., 2021; Dong et al., 2022; Kim et al., 2020; Zhang et al., 2021; Lin et al., 2021
Sorafenib	35.23 (28.18–42.28)	Wen et al., 2021, Meng et al., 2022, Gu et al., 2019	762.46 (609.97–914.95)	Wen et al., 2021, Dong et al., 2022, Kim et al., 2020, Zhang et al., 2021, Lin et al., 2021
Disutility of AEs in each group (grade ≥3)				
Camr-rivo	0.07 (0.05–0.08)	Liu et al., 2021b; Telford et al., 2019; Hoerger et al., 2010; Shao et al., 2022; Kim et al., 2020	0.07 (0.053–0.079)	Liu et al., 2021a; Telford et al., 2019; Hoerger et al., 2010; Shao et al., 2022; Kim et al., 2020
Sorafenib	0.038 (0.03–0.05)	Liu et al., 2021a; Liu et al., 2021b, Telford et al., 2019, Hoerger et al., 2010, Shao et al., 2022; Kim et al., 2020)	0.038 (0.03–0.05)	Liu et al., 2021a; Telford et al., 2019; Hoerger et al., 2010; Shao et al., 2022; Kim et al., 2020
Utility				
Progression-free survival	0.76 (0.61–0.91)	Wen et al. (2021)	0.76 (0.61–0.91)	Wen et al. (2021)
Progressive disease	0.68 (0.54–0.82)	Wen et al. (2021)	0.68 (0.54–0.82)	Wen et al. (2021)
Discount rate	0.05 (0.04–0.06)	Chinese Pharmaceutical Association, 2020	0.03 (0.02–0.04)	Chinese Pharmaceutical Association, 2020
Survival inputs				
Camr-rivo group OS Survival Model	Shape = 1.400 Scale = 29.632QA	Qin et al. (2023)	—	—
Camr-rivo group PFS Survival Model	Shape = 1.632 Rate = 0.215	Qin et al. (2023)	—	—
Sorafenib group OS Survival Model	Shape = 1.617 Rate = 0.079	Qin et al. (2023)	—	—
Sorafenib group PFS Survival Model	Shape = 2.094 Rate = 0.461	Qin et al. (2023)	—	—

^a^ Values are taken as ±20% of the baseline value.

Abbreviations: US, United States; CT, computed tomography; MRI, magnetic resonance imaging; AE, adverse event.

### 3.4 Sensitivity analyses

In this study, one-way sensitivity analyses and probabilistic sensitivity analyses (PSAs) were conducted to explore the uncertainty of the results and the robustness of the model. The range of variation in parameters is listed in Table 1. In the absence of upper and lower limit values, the values were calculated as ±20% of the parameters. In PSA, Monte Carlo simulations were performed with 1,000 iterations, sampling from different distribution parameters simultaneously (gamma distribution was selected for cost-related parameters, and beta distribution was adopted for utility value parameters and probabilities). The results are illustrated by tornado diagrams, cost-utility acceptability curves, and ICUR scatter plots.

### 3.5 Scenario analyses

We conducted a scenario analysis with different prices of camr-rivo from the US payers’ perspective to explore their influence on the ICUR. Price reductions of 10%, 20%, and 30% were made for camr-rivo, and the price of each drug was also reduced separately.

## 4 Results

### 4.1 Base-case analyses

Reconstructed Kaplan–Meier curves and extrapolated survival curves for OS and PFS are shown in Supplementary Figure S1, S2. Based on the model results, compared with sorafenib, camr-rivo provided an additional 0.34 QALY and an incremental cost of $4,762.10 and $92,700.49 in China and the US, respectively, thus yielding ICURs of $14,174.40/QALY and $272,852.59/QALY in these two countries, respectively (Table 2). Compared to the set WTP, camr-rivo is more economical than sorafenib in China but not in the US.

**TABLE 2:** Base-case analyses (China, WTP = $36,627.25/QALY; US, WTP = $150,000.00/QALY).

Strategy	Cost ($)	QALYs	ICUR ($/QALY)
Chinese healthcare system perspective			
Camr-rivo	26,362.55	1.60	14,174.40
Sorafenib	21,600.45	1.26	NA
US payers’ perspective			
Camr-rivo	289,785.47	1.60	272,852.59
Sorafenib	197,084.99	1.26	NA

Abbreviations: Camr-rivo, camrelizumab–rivoceranib; QALYs, quality-adjusted life years; ICUR, incremental cost-utility ratio.

### 4.2 Sensitivity analyses

**FIGURE 2:**
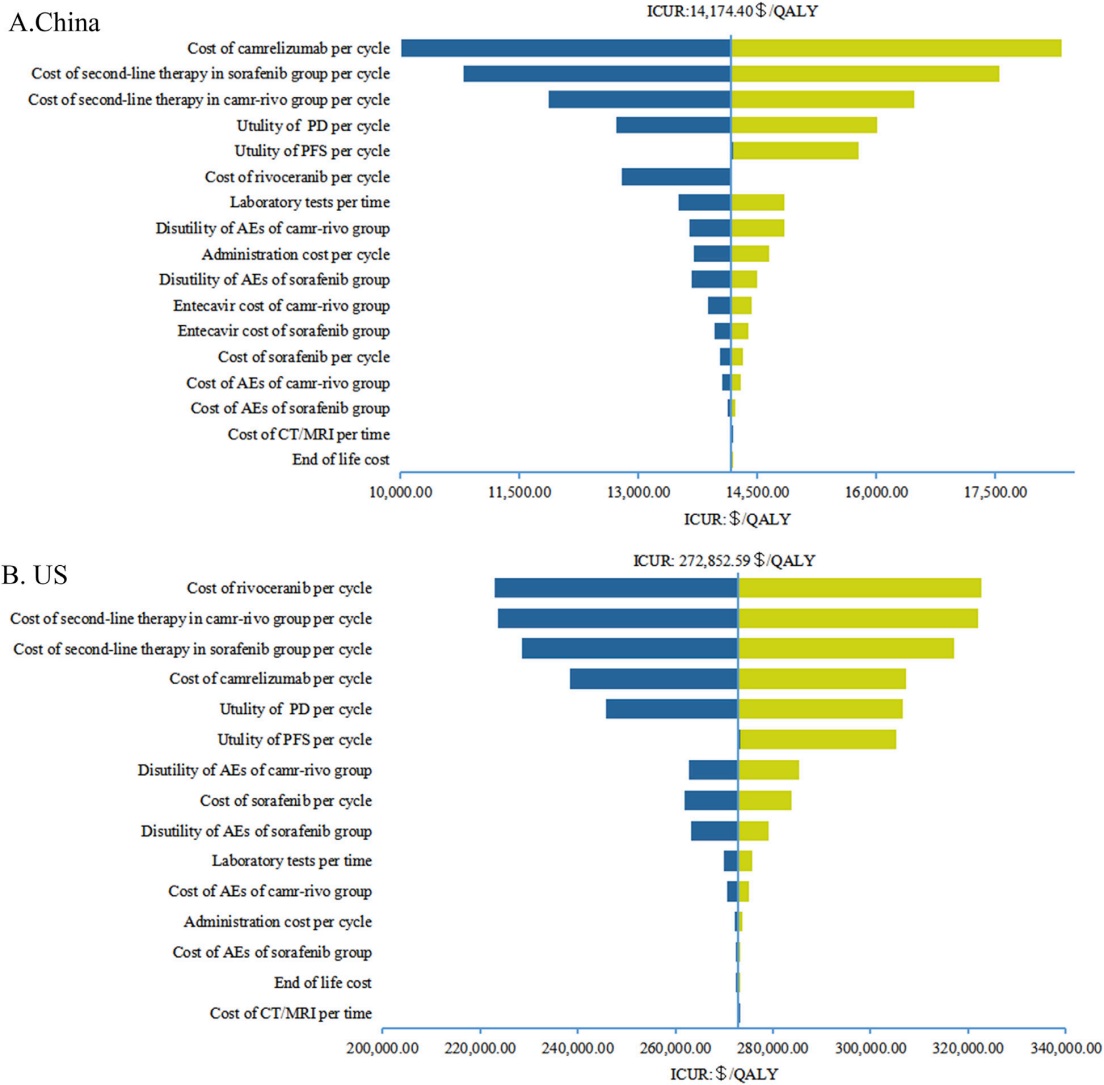
Tornado diagrams of one-way sensitivity analyses in China **(A)** and in the US **(B)**. AEs, adverse events; PFS, progression-free survival; PD, progressive disease; CT, computed tomography; MRI, magnetic resonance imaging; QALY, quality-adjusted life-year; ICUR, incremental cost-utility ratios.

**FIGURE 3:**
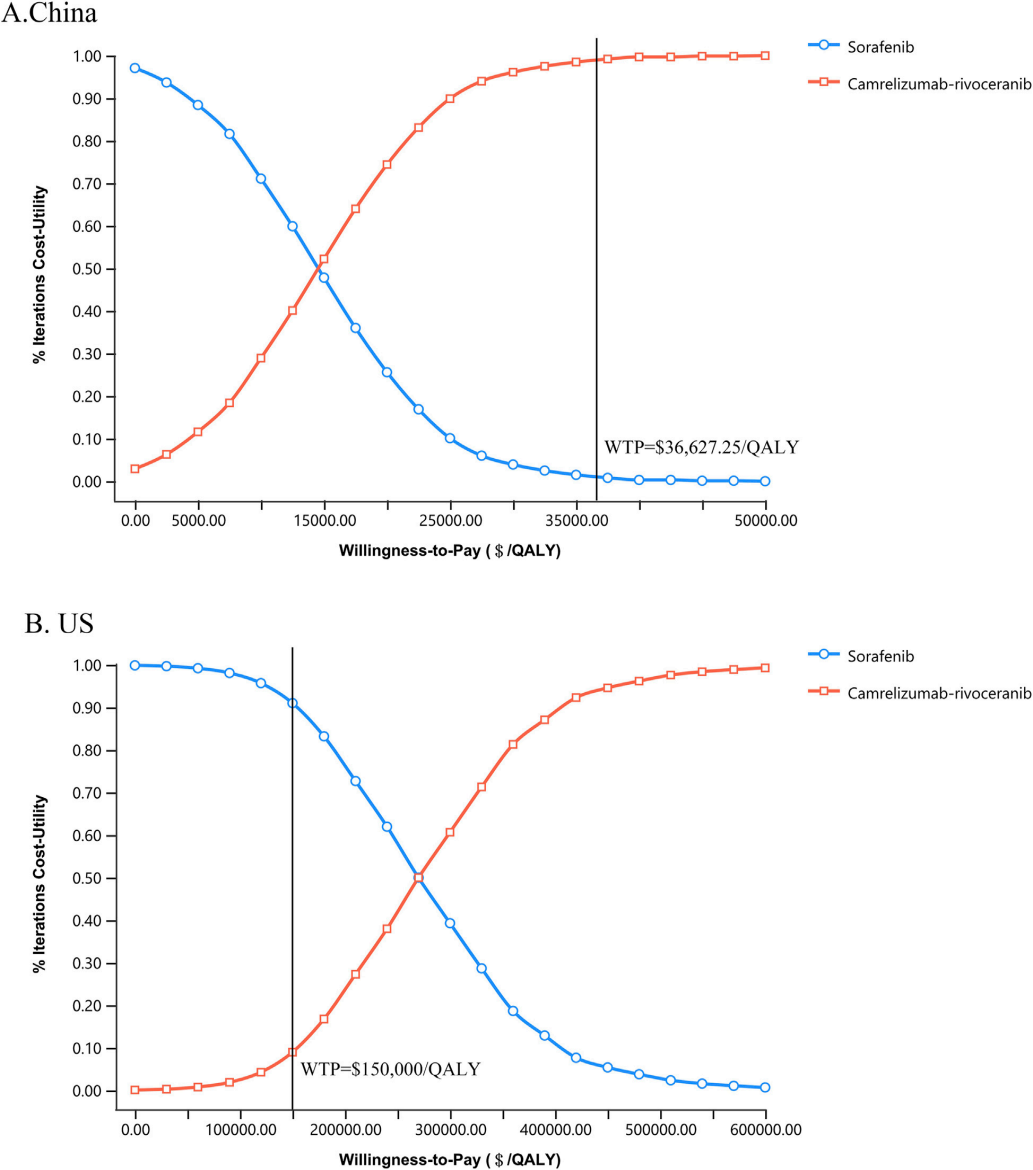
Cost-utility acceptability curves of camr-rivo group and sorafeinib group in China **(A)** and the US **(B)**. QALY, quality-adjusted life-year; WTP, willingness-to-pay.

The results of one-way sensitivity analyses are illustrated in the tornado diagrams (Figure 2). The cost of rivoceranib per cycle in the camr-rivo group was the most influential variable for ICUR from the US payers’ perspective, followed by the cost of second-line therapy and the cost of camrelizumab per cycle. The results in China were substantially sensitive to the cost of camrelizumab, followed by the cost of second-line therapy per cycle in both groups. However, changes in the key model parameters within a reasonable range did not affect the results. PSA results in cost–utility acceptability curves (Figure 3) showed that the probabilities of camr-rivo being cost-efficient were 99.4% and 9.5%, respectively, at the WTP thresholds of $36,627.25/QALY and $150,000.00/QALY in China and the US, respectively. The ICUR scatter points were located above the first quadrant of the axis, indicating that camr-rivo resulted in a better QALY but higher costs. From a Chinese perspective, most scatter points were below the WTP, indicating that camr-rivo was more economical than sorafenib. In contrast, in the US model, the scatter of results was mainly distributed over WTP, indicating that camr-rivo was not cost-efficient compared to sorafenib. Most of the scatter points in the figure are also within the 95% confidence interval (Supplementary Figure S3).

### 4.3 Scenario analyses

From the US payers’ perspective, the results showed that camr-rivo was economical with an ICUR below the WTP threshold when the prices of camrelizumab and rivoceranib were simultaneously reduced by 30% of their original prices. If the price of rivoceranib remained unchanged, camrelizumab would need to be reduced by approximately 70% to make the regimen economical, whereas if it remained at its original price, rivoceranib would need to be reduced by 50% to make the regimen economical (Table 3).

**TABLE 3:** Scenario analyses (US, WTP = $150,000.00/QALY).

Discount drug	Both camrelizumab and rivoceranib			Rivoceranib	Camrelizumab
Price reduction	30%	20%	10%	50%	72%
Increased cost ($)	49,761.16	64,074,21	78,387.38	50,432.26	50,512.34
ICUR ($/QALY)	146,465.16	188,594.81	230,723.70	148,441.23	148,676.93

Abbreviations: ICUR, incremental cost–utility ratio; QALY, quality-adjusted life years.

## 5 Discussion

HCC poses a major global health challenge, affecting millions of individuals worldwide. Fortunately, with the in-depth research on PD-1/PD-L1 inhibitors and targeted drugs, an increasing number of combination therapy strategies have been widely used in the first-line treatment of unresectable or advanced HCC, which can effectively improve the PFS and OS of patients with HCC. Nonetheless, HCC also has a negative impact on economic growth, and there remains a heavy economic burden on patients with HCC, accounting for 24.1% and 20.8% of the global economic burden of cancer in China and the US, respectively (Chen et al., 2023). Notably, China and the US face the greatest economic costs of cancer, which represents nearly half of the worldwide economic burden. The estimated global economic cost of cancer from 2020 to 2050 will reach $25.2 trillion in international dollars, with HCC ranking fourth in terms of economic expenditure.

Moreover, PD-1/PD-L1 inhibitors are expensive; despite the promising efficacy demonstrated by the combination of atezolizumab plus bevacizumab in previous studies for first-line treatment of unresectable or advanced HCC, this regimen was hardly economical for HCC patients both in China and the US (Hou and Wu, 2020; Patel et al., 2021). Neither nivolumab nor pembrolizumab is less economical than sorafenib in the first- or second-line treatment of advanced HCC(Chiang et al., 2021; Shu et al., 2023). If a treatment is to be deemed economical, it must have better efficacy and lower costs within a health and economic assessment model; therefore, it is necessary to evaluate the economics of new treatment strategies.

This study analyzed the cost–utility of camr-rivo versus sorafenib from the perspectives of the Chinese healthcare system and US payers. The results showed that the incremental QALY was 0.34. The ICUR of camr-rivo compared to sorafenib as the first-line treatment for unresectable or advanced HCC in China was $14,174.40/QALY, which was lower than the WTP threshold ($36,627.25/QALY), suggesting that camr-rivo is a more economical treatment option than sorafenib in China. This may be due to the significant decrease in the price of camrelizumab, which has been included in medical insurance in recent years, resulting in a lower price for PD-1/PD-L1 inhibitors that have been approved for marketing. This has greatly reduced the economic burden on Chinese patients and made camr-rivo economical. In addition, the results of the one-way sensitivity analysis also showed that the results of the Chinese model were most affected by the price of camrelizumab, but this did not fundamentally change the results.

From the US payers’ perspective, the incremental cost per QALY obtained with camr-rivo versus sorafenib was $272,852.59, which is much higher than the WTP threshold. Sensitivity analyses indicated that the ICUR in the US was most sensitive to the price fluctuation of rivoceranib, followed by the cost of second-line treatment, cost of camrelizumab, and utility value of PD and PFS. However, since camrelizumab and rivoceranib were not available in the US, the cost of camrelizumab per cycle was equal to the cost of atezolizumab in China, and the price of rivoceranib was replaced by the price of lenvatinib in the US. The price of rivoceranib was calculated by multiplying the price of lenvatinib in the US by the price ratio of lenvatinib and rivoceranib in China. This may overestimate the cost of camr-rivo, resulting in it not being economical in the US. Therefore, we conducted multiple scenario analyses on the cost of camr-rivo, and the results showed that when both the prices of camrelizumab and rivoceranib are reduced by approximately 30%, camr-rivo will be more economical than sorafenib in the US.

To the best of our knowledge, the marketing application of camr-rivo as a first-line treatment for advanced or unresectable HCC has been accepted by the US Food and Drug Administration, indicating that camr-rivo is likely to be marketed in the US. However, no pharmacoeconomic studies have evaluated the combination of camr-rivo from the US perspective. Liu et al. (2022) reported that camr-rivo was more economical than sorafenib from the Chinese payer’s perspective, which was consistent with our results. However, the study indirectly compared the cost-effectiveness of 15 first-line treatment options for advanced HCC using HR values and assumed that the patients only received regorafenib as a second-line treatment after disease progression. In contrast, we extracted survival data directly from the study for head-to-head comparisons, which was more accurate and precise. Additionally, according to clinical trials (CARES-310), there are many second-line treatment options for patients after disease progression, including targeted therapy, chemotherapy, and immunotherapy. We included multiple options for second-line treatment that were more closely aligned with the actual situation of the clinical treatment. Moreover, our study was conducted from the perspective of the Chinese healthcare system and US payers, which has important implications for both developing and developed countries.

We conducted PSM to evaluate the cost–utility of camr-rivo versus sorafenib. In recent years, many studies have applied PSM for cancer pharmacoeconomic evaluation. PSM directly uses a set of survival curves to determine the number or proportion of patients in each status (status membership), which does not need to calculate the transition probability and avoids unnecessary model assumptions affecting the study results. Bullement et al. (2019) showed that PSM was the most commonly used model (54%), followed by status transition models (including Markov models) (41%) from 2013 to 2018 among nearly 100 oncology drug evaluation reports from the National Institute for Health and Care Excellence (NICE). Compared with the Markov model, PSM was easier to construct with a simple and clear structure, and no additional assumptions were made (Rui et al., 2021). Goeree et al. (2016) discovered that, based on accurate utilization and validation of the model, both PSM and the standard Markov model yielded comparable expected outcomes, indicating that modeling accuracy was unaffected by the model type itself. Furthermore, a highlight of our study is the consideration of antiviral therapy in patients with HCC, for which hepatitis B virus (HBV) infection was the most noteworthy risk factor for HCC. HBV infection accounted for 76% and 73% of all patients in the camr-rivo and sorafenib groups, respectively. Patients receiving systemic antitumor therapy are at a higher risk of HBV reactivation. Guidelines recommend first-line antiviral drugs, such as entecavir or tenofovir, for the treatment and prevention of HBV reactivation. Therefore, we assumed that patients were given entecavir along with systemic therapy, and the antiviral costs were calculated in our study. This aspect was not addressed in the study by Zhao et al. (2024). Our comprehensive consideration of costs gives our findings a higher degree of credibility.

Our study has some limitations. First, the model incorporated utility values from published articles pertaining to HCC and its corresponding disease status, which exhibited variability within a given range without significantly altering the qualitative outcomes. Second, only the treatment cost and disutility values of grade ≥ 3 AEs were included in the model, and the effect of grade 1–2 AEs was ignored. However, one-way sensitivity analysis showed that the treatment cost and disutility values of AEs had little effect on the results. In addition, because camrelizumab and rivoceranib are not available in the US, the specific price information obtained is challenging. We indirectly assumed drug costs based on the prices of atezolizumab and lenvatinib, which may have influenced the calculation of the total cost of camr-rivo therapy from the US payers’ perspective and biased the results. Nevertheless, according to the sensitivity analysis, this did not affect the study’s results when the costs of camrelizumab and rivoceranib fluctuated within this range. Furthermore, the WTP threshold set in this study was $150,000/QALY; however, research by Neumann Kim (2023) shows that the $100,000/QALY threshold remains the most frequently cited benchmark in cost-effectiveness analyses in the US. However, their analysis also revealed important background differences: cancer-related studies were 2.22 times more likely to adopt the $150,000/QALY threshold compared to non-cancer studies (95% CI:1.70-2.90) (117/658 [17.8%] vs. 159/2618 [6.1%]). This is consistent with our logic for testing a higher threshold as HCC therapies often face higher willingness to pay considerations given the high unmet medical need and life-threatening nature of advanced liver cancer treatment. Importantly, as illustrated in Figure 3, our base-case conclusions remain robust across both thresholds.

## 6 Conclusion

From the Chinese healthcare system’s perspective, camrelizumab plus rivoceranib is likely to be more cost-effective than sorafenib as a first-line treatment for patients with unresectable or advanced HCC. From the US payers’ perspective, camrelizumab plus rivoceranib is unlikely to be considered economical at the WTP threshold of $150,000.00/QALY. However, simultaneously reducing the prices of camrelizumab and rivoceranib to 70% of their original prices could make camrelizumab plus rivoceranib regimen more economical than sorafenib alone in the US. This study can serve as a reference point for camrelizumab and rivoceranib pricing within the US market. Therefore, lowering the price and bundle sales of camrelizumab and rivoceranib may be an effective and economical strategy.

## Data Availability

The original contributions presented in the study are included in the article/Supplementary Material; further inquiries can be directed to the corresponding author.
