# Supplementary material for: Camrelizumab plus rivoceranib versus sorafenib as first-line therapy for patients with unresectable hepatocellular carcinoma: a cost–utility analysis in China and the United States
Source: Front Pharmacol. 2025 May 1;16:1404389. doi: 10.3389/fphar.2025.1404389 (PMC12078126; doi:10.3389/fphar.2025.1404389)
Supplement: Supplementary file 1 [file DataSheet1.docx]

Supplementary Material

Supplementary Figure 1. The reconstructed Kaplan-Meier PFS and OS curves of the camr-rivo group and the sorafenib group according to the CARES-310 Trial.

Supplementary Figure 2. Extrapolated the survival curves.

Supplementary Figure 3. Cost-utility scatter plots of the camr-rivo group and the sorafenib group.

Supplementary Table 1. The proportion of patients receiving each second-line regimenand the cost of second-line therapies in China and the United States

Supplementary Table 2. The specific course of second-line treatment.

Supplementary Table 3. AIC values and BIC values from each survival model.

Supplementary Table 4. The dis-utility and the treatment cost of adverse events≥3 grade.

Supplementary Table 5. The probability of adverse events (Grade ≥3).

Supplementary Table 6. CHEERS 2022 checklist.


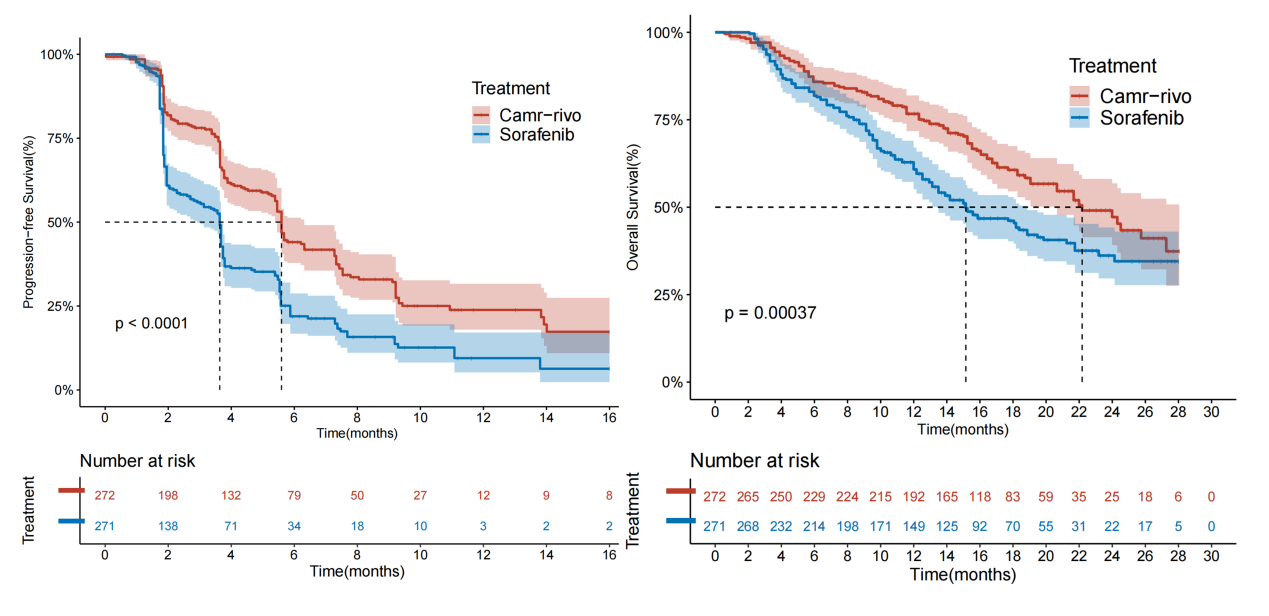


Supplementary Figure 1. The reconstructed Kaplan-Meier PFS and OS curves of the camr-rivo group and the sorafenib group according to the CARES-310 Trial.

Abbreviations: PFS, Progression-free survival; OS,Overall survival; camr-rivo, camrelizumab-rivoceranib.


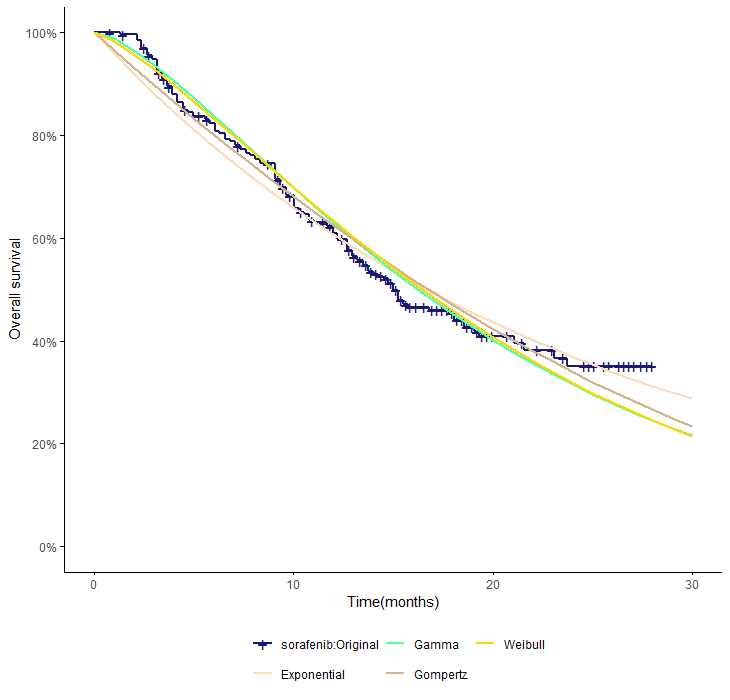

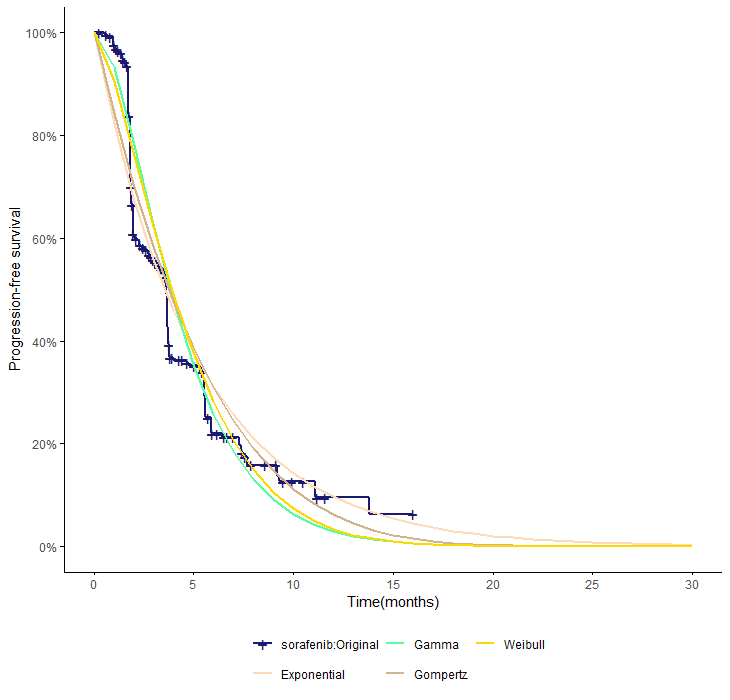

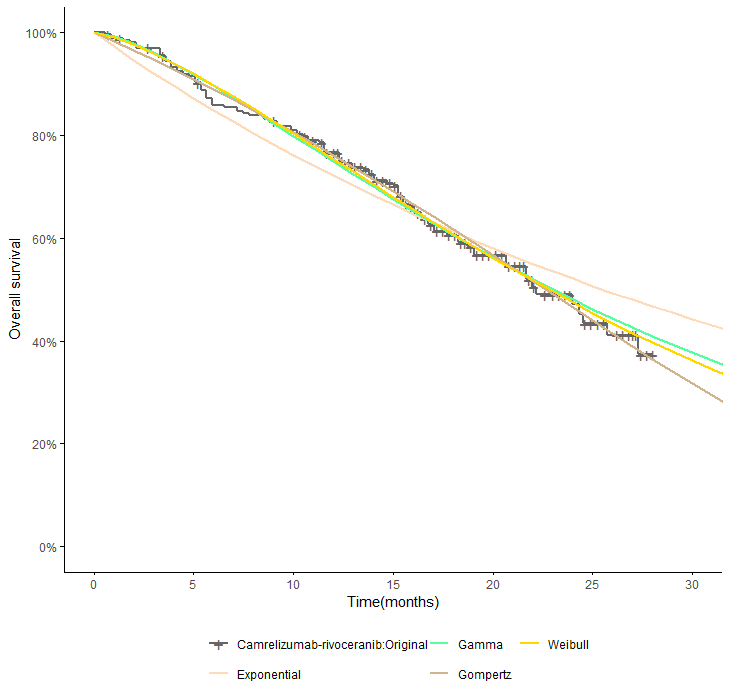

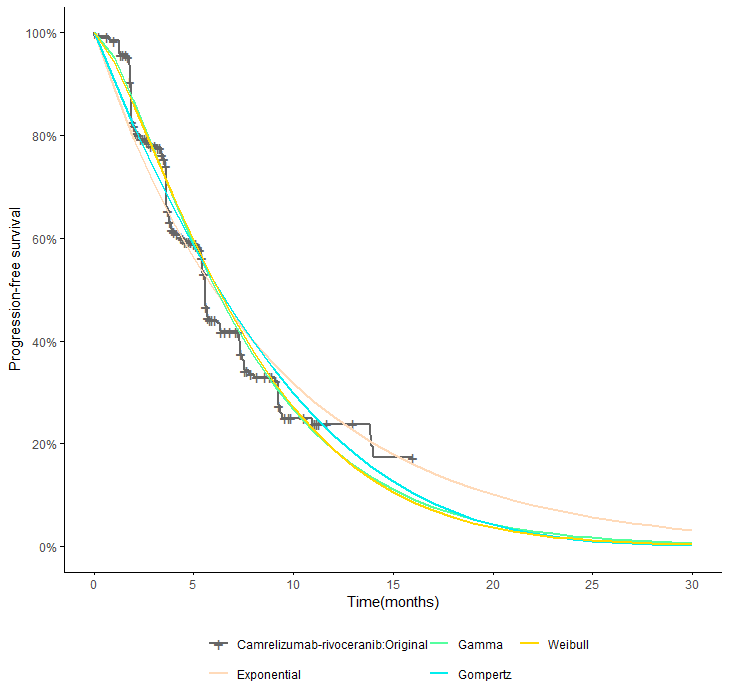

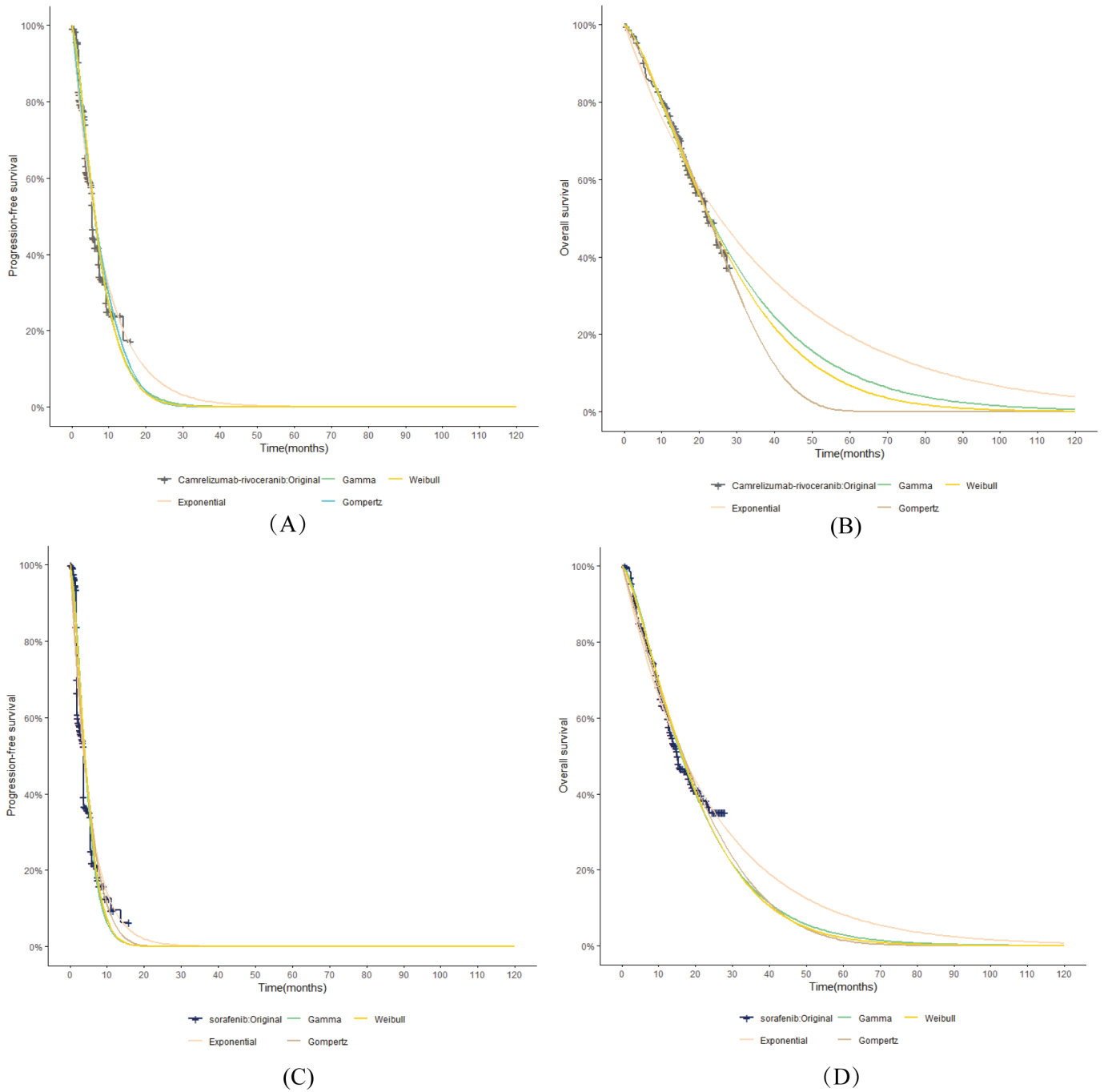


Supplementary Figure 2. Extrapolated survival curves. (A) PFS curve and fitting curve of camr-rivo group (B) OS curve and fitting curve of camr-rivo group (C) PFS curve and fitting curve of sorafenib group (D) OS curve and fitting curve of sorafenib group.

Abbreviations: PFS, Progression-free survival; OS,Overall survival; camr-rivo, camrelizumab-rivoceranib.


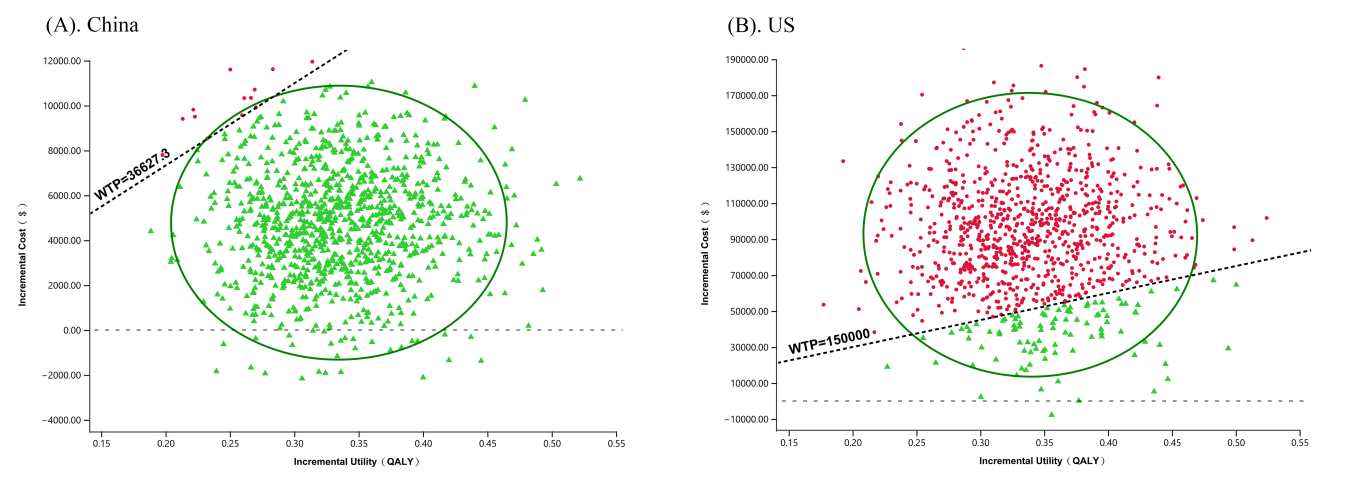


Supplementary Figure 3.Cost-utility scatter plots, Camr-rivo group vs. Sorafenib group;

Abbreviations: Camr-rivo, Camrelizumab-rivoceranib.

Supplementary Table 1. The proportion of patients receiving each second-line regimen and the cost of second-line therapies in China and the United States

|  | Propotion | Cost（$/per cycle） | Cost（$/per cycle） | References |
| --- | --- | --- | --- | --- |
| Camr-rivo group | | | | |
| Lenvatinib | 0.38 | 67.53 | 1460.58 | [1,2] |
| Camrelizumab | 0.09 | 22.09 | 185.10 | [1] |
| Sorafenib | 0.29 | 23.39 | 1763.67 | [1,3] |
| Regorafenib | 0.09 | 62.11 | 394.67 | [1,4] |
| Sintilimab | 0.08 | 10.58 | 158.66 | [1,3] |
| Rivoceranib | 0.04 | 5.39 | 116.47 | [1,2] |
| Capecitabine | 0.04 | 0.63 | 70.20 | [1,3] |
| Total cost |  | China：$191.72/cycle | The US：$4149.34/cycle |  |
| Sorafenib group | | | | |
| Lenvatinib | 0.23 | 59.75 | 1292.21 | [1,2] |
| Camrelizumab | 0.26 | 92.14 | 772.01 | [1] |
| Sorafenib | 0.06 | 6.58 | 496.48 | [1,3] |
| Regorafenib | 0.13 | 125.60 | 798.10 | [1,4] |
| Sintilimab | 0.14 | 28.09 | 421.10 | [1,3] |
| Rivoceranib | 0.15 | 30.17 | 652.61 | [1,2] |
| Capecitabine | 0.04 | 0.93 | 103.51 | [1,3] |
| Total cost |  | China：$343.27/cycle | The US：$4536.01/cycle |  |

Abbreviations: Camr-rivo, Camrelizumab-rivoceranib.

Supplementary Table 2. The specific course of second-line treatment.

| Drug Name | detailed medication regimen |
| --- | --- |
| Lenvatinib | 12mg/day, 28 days, orally |
| Camrelizumab | 200mg/ two weeks, intravenously |
| Sorafenib | 400mg twice daily, 28 days, orally |
| Regorafenib | 160mg/day for the first 3 weeks, with a 28-day course as one treatment cycle, orally |
| Sintilimab | 200mg, once every 3 weeks, intravenously |
| Rivoceranib | 250 mg/day, 28 days, orally |
| Capecitabine | 1250mg/m², twice daily (once in the morning and once in the evening), medication is taken continuously for 2 weeks followed by a 1-week break,with a 21-day course as one treatment cycle, orally |

Supplementary Table 3. AIC values and BIC values from each survival model.

| Distribution | Camr-rivo group | | | | Sorafenib group | | | |
| --- | --- | --- | --- | --- | --- | --- | --- | --- |
|  | AIC-PFS | BIC-PFS | AIC-OS | BIC-OS | AIC-PFS | BIC-PFS | AIC-OS | BIC-OS |
| Exponential | 989.51 | 993.11 | 1015.27 | 1018.88 | 956.83 | 960.43 | 1241.43 | 1245.03 |
| Gamma | 968.63 | 975.84 | 1003.89 | 1011.10 | 905.98 | 913.19 | 1223.83 | 1231.03 |
| Gompertz | 987.25 | 994.46 | 1005.86 | 1013.08 | 954.52 | 961.73 | 1240.52 | 1247.72 |
| Weibull | 972.81 | 980.02 | 1003.69 | 1010.90 | 924.19 | 931.39 | 1228.40 | 1235.60 |

Abbreviations: Camr-rivo, Camrelizumab-rivoceranib; AIC, Akaike information criterion; BIC, Bayesian information criterion; KM, Kaplan-Meier; OS, overall survival; PFS, progression-free survival.

Supplementary Table 4. The dis-utility and the treatment cost of adverse events ≥3 grade.

| Dis-utility | Baseline value | Range | Distribution | References |
| --- | --- | --- | --- | --- |
| Hypertension | 0.05 | 0.04-0.06 | Beta | [5] |
| Aspartate aminotransferase increased | 0 | 0 | Beta | [6] |
| Proteinuria | 0.01 | 0.008-0.012 | Beta | [7] |
| Alanine aminotransferase increased | 0.05 | 0.04-0.06 | Beta | [6] |
| Platelet count decreased | 0.11 | 0.09-0.13 | Beta | [8] |
| Blood bilirubin increased | 0 | 0 | Beta | [9] |
| Palmar–plantar erythrodysesthesia syndrome | 0.116 | 0.09-0.14 | Beta | [9] |
| Dis-utility | Baseline value | Range | Distribution | References |
| Neutrophil count decreased | 0.2 | 0.16-0.24 | Beta | [8] |
| Diarrhea | 0.12 | 0.096-0.144 | Beta | [9] |
| Costs per event ($/once), in China | | | | |
| Hypertension | 1.36 | 1.09-1.63 | Gamma | [10] |
| Aspartate aminotransferase increased | 87.93 | 70.34-10.55 | Gamma | [10] |
| Proteinuria | 103.77 | 83.01-124.52 | Gamma | [11] |
| Alanine aminotransferase increased | 87.93 | 70.34-105.51 | Gamma | [10] |
| Platelet count decreased | 1061.79 | 849.43-1274.15 | Gamma | [10] |
| Blood bilirubin increased | 114.35 | 91.48-137.21 | Gamma | [10] |
| Palmar–plantar erythrodysesthesia syndrome | 33.76 | 27.00-40.50 | Gamma | [10] |
| Neutrophil count decreased | 451.45 | 361.16-541.74 | Gamma | [12] |
| Diarrhea | 3.30 | 2.64-3.96 | Gamma | [10] |
| Costs per event ($/once), in the US | | | | |
| Hypertension | 34.96 | 27.97-41.95 | Gamma | [10] |
| Aspartate aminotransferase increased | 6384.62 | 5107.70-7661.55 | Gamma | [9] |
| Proteinuria | 3138.59 | 2510.87-3766.30 | Gamma | [13] |
| Alanine aminotransferase increased | 452.08 | 361.67-542.50 | Gamma | [14] |
| Platelet count decreased | 1092.06 | 873.65-1310.48 | Gamma | [13] |
| Blood bilirubin increased | 1026.11 | 820.89-1231.33 | Gamma | [9] |
| Palmar–plantar erythrodysesthesia syndrome | 107.77 | 86.21-129.32 | Gamma | [10] |
| Neutrophil count decreased | 12317.49 | 9853.99-14780.99 | Gamma | [15] |
| Diarrhea | 17.52 | 14.02-21.03 | Gamma | [10] |

Supplementary Table 5. The probability of adverse events (Grade ≥3).

| Probability of adverse events | Sorafenib group | Camr-rivo group | Distribution | References |
| --- | --- | --- | --- | --- |
| Hypertension | 0.15 | 0.38 | Beta | [16] |
| Aspartate aminotransferase increased | 0.05 | 0.16 | Beta | [16] |
| Proteinuria | 0.02 | 0.06 | Beta | [16] |
| Alanine aminotransferase increased | 0.03 | 0.14 | Beta | [16] |
| Platelet count decreased | 0.01 | 0.11 | Beta | [16] |
| Blood bilirubin increased | 0.01 | 0.09 | Beta | [16] |
| Palmar–plantar erythrodysesthesia syndrome | 0.15 | 0.12 | Beta | [16] |
| Neutrophil count decreased | 0.02 | 0.05 | Beta | [16] |
| Diarrhea | 0.02 | 0.02 | Beta | [16] |

Abbreviations: AEs, Adverse events; Camr-rivo, Camrelizumab-rivoceranib.

Supplementary Table 6. CHEERS 2022 checklist.

| Topic | Item. | Guidance for Reporting. | Reported in section |
| --- | --- | --- | --- |
| Title |  |  |  |
|  | 1 | Camrelizumab plus rivoceranib versus sorafenib as first-line therapy for patients with unresectable or advanced hepatocellular carcinoma: A cost-utility analysis in China and the United states. | Page1 |
| Abstract |  |  |  |
|  | 2 | Provide a structured summary that highlights context, key methods, results, and alternative analyses. | Page1 |
| Introduction |  |  |  |
| Background and objectives | 3 | Give the context for the study, the study question, and its practical relevance for decision making in policy or practice. | Page1 and Page2 |
| Methods |  |  |  |
| Health economic analysis plan | 4 | Indicate whether a health economic analysis plan was developed and where available. | Not Applicable |
| Study population | 5 | Describe characteristics of the study population (such as age range, demographics, socioeconomic, or clinical characteristics). | Page2 and Page3 |
| Setting and location | 6 | Provide relevant contextual information that may influence findings. | Page4 |
| Comparators | 7 | Describe the interventions or strategies being compared and why chosen. | Page3 |
| Perspective | 8 | State the perspective(s) adopted by the study and why chosen. | Page2 |
| Time horizon | 9 | State the time horizon for the study and why appropriate. | Page3 |
| Discount rate | 10 | Report the discount rate(s) and reason chosen. | Page3 |
| Selection of outcomes | 11 | Describe what outcomes were used as the measure(s) of benefit(s) and harm(s). | Page4 |
| Measurement of outcomes | 12 | Describe how outcomes used to capture benefit(s) and harm(s) were measured. | Page4 |
| Valuation of outcomes | 13 | Describe the population and methods used to measure and value outcomes. | Page4 |
| Measurement and valuation of resources and costs | 14 | Describe how costs were valued. | Page4 |
| Currency, price date, and conversion | 15 | Report the dates of the estimated resource quantities and unit costs, plus the currency and year of conversion. | Page4 |
| Rationale and description of model | 16 | If modelling is used, describe in detail and why used. Report if the model is publicly available and where it can be accessed. | Not Applicable |
| Analytics and assumptions | 17 | Describe any methods for analysing or statistically transforming data, any extrapolation methods, and approaches for validating any model used. | Page3 |
| Characterising heterogeneity | 18 | Describe any methods used for estimating how the results of the study vary for subgroups. | Not Applicable |
| Characterising distributional effects | 19 | Describe how impacts are distributed across different individuals or adjustments made to reflect priority populations. | Page4 |
| Characterising uncertainty | 20 | Describe methods to characterise any sources of uncertainty in the analysis. | Page4 |
| Approach to engagement with patients and others affected by the study | 21 | Describe any approaches to engage patients or service recipients, the general public, communities, or stakeholders (such as clinicians or payers) in the design of the study. | Not Applicable |
| Results |  |  |  |
| Study parameters | 22 | Report all analytic inputs (such as values, ranges, references) including uncertainty or distributional assumptions. | Page5 |
| Summary of main results | 23 | Report the mean values for the main categories of costs and outcomes of interest and summarise them in the most appropriate overall measure. | Page5 |
| Effect of uncertainty | 24 | Describe how uncertainty about analytic judgments, inputs, or projections affect findings. Report the effect of choice of discount rate and time horizon, if applicable. | Page5 |
| Effect of engagement with patients and others affected by the study | 25 | Report on any difference patient/service recipient, general public, community, or stakeholder involvement made to the approach or findings of the study | Not Applicable |
| Discussion |  |  |  |
| Study findings, limitations, generalisability, and current knowledge | 26 | Report key findings, limitations, ethical or equity considerations not captured, and how these could affect patients, policy, or practice. | Page7 |
| Other relevant information |  |  |  |
| Source of funding | 27 | Describe how the study was funded and any role of the funder in the identification, design, conduct, and reporting of the analysis | Page8 |
| Conflicts of interest | 28 | Report authors conflicts of interest according to journal or International Committee of Medical Journal Editors requirements. | Page8 |

References

[1] yaozh.com. <https://db.yaozh.com/yaopinzhongbiao>. Accessed December 15, 2023.

[2] Kim JJ, McFarlane T, Tully S, Wong WWL. Lenvatinib Versus Sorafenib as First-Line Treatment of Unresectable Hepatocellular Carcinoma: A Cost-Utility Analysis. Oncologist. 2020;25(3):e512-e519.

[3] Micromedex Solutions. Red book online. [http://www.micromedexsolutions.com](http://www.micromedexsolutions.com/) Accessed December 15, 2023.

[4] Parikh ND, Singal AG, Hutton DW. Cost effectiveness of regorafenib as second-line therapy for patients with advanced hepatocellular carcinoma. Cancer. 2017;123(19):3725-3731.

[5] Liu L, Bai H, Wang C, et al. Efficacy and Safety of First-Line Immunotherapy Combinations for Advanced NSCLC: A Systematic Review and Network Meta-Analysis. *J Thorac Oncol*. 2021;16(7):1099-1117.

[6] Telford C, Bertranou E, Large S, Phelps H, Ekman M, Livings C. Cost-Effectiveness Analysis of Fulvestrant 500 mg in Endocrine Therapy-Naïve Postmenopausal Women with Hormone Receptor-Positive Advanced Breast Cancer in the UK. Pharmacoecon Open. 2019;3(4):559-570.

[7] THOMAS J. HOERGER, JOHN S. WITTENBORN, JOEL E. SEGEL, et al. A Health Policy Model of CKD: 2. The Cost-Effectiveness of Microalbuminuria Screening[J]. American Journal of Kidney Diseases, 2010, 55 (3): 463-473.

[8] Shao T, Ren Y, Zhao M, Tang W. Cost-effectiveness analysis of camrelizumab plus chemotherapy as first-line treatment for advanced squamous NSCLC in China. Front Public Health. 2022;10:912921.

[9] Kim JJ, McFarlane T, Tully S, Wong WWL. Lenvatinib Versus Sorafenib as First-Line Treatment of Unresectable Hepatocellular Carcinoma: A Cost-Utility Analysis. Oncologist. 2020;25(3):e512-e519.

[10] Wen F, Zheng H, Zhang P, Liao W, Zhou K, Li Q. Atezolizumab and bevacizumab combination compared with sorafenib as the first-line systemic treatment for patients with unresectable hepatocellular carcinoma: A cost-effectiveness analysis in China and the United states. Liver Int. 2021;41(5):1097-1104.

[11] Meng R, Zhang X, Zhou T, Luo M, Qiu Y. Cost-effectiveness analysis of donafenib versus lenvatinib for first-line treatment of unresectable or metastatic hepatocellular carcinoma. Expert Rev Pharmacoecon Outcomes Res. 2022;22(7):1079-1086.

[12] Gu X, Zhang Q, Chu YB, et al. Cost-effectiveness of afatinib, gefitinib, erlotinib and pemetrexed-based chemotherapy as first-line treatments for advanced non-small cell lung cancer in China. Lung Cancer. 2019;127:84-89.

[13]Zhang X, Wang J, Shi J, Jia X, Dang S, Wang W. Cost-effectiveness of Atezolizumab Plus Bevacizumab vs Sorafenib for Patients With Unresectable or Metastatic Hepatocellular Carcinoma. JAMA Netw Open. 2021;4(4):e214846.

[14] Dong L, Lin S, Zhong L, et al. Evaluation of Tucatinib in HER2-Positive Breast Cancer Patients With Brain Metastases: A United States-Based Cost-Effectiveness Analysis. Clin Breast Cancer. 2022;22(1):e21-e29.

[15] Lin S, Luo S, Gu D, et al. First-Line Durvalumab in Addition to Etoposide and Platinum for Extensive-Stage Small Cell Lung Cancer: A U.S.-Based Cost-Effectiveness Analysis. Oncologist. 2021;26(11):e2013-e2020.

[16] Qin S, Chan SL, Gu S, et al. Camrelizumab plus rivoceranib versus sorafenib as first-line therapy for unresectable hepatocellular carcinoma (CARES-310): a randomised, open-label, international phase 3 study. *Lancet*. 2023;402(10408):1133-1146.
